# Determinants of COVID-19 vaccine hesitancy among healthcare personnel in a secondary-level health facility in Ghana

**DOI:** 10.1371/journal.pgph.0004980

**Published:** 2025-09-18

**Authors:** Gloria Addobea Kumi, Mawuli Gohoho, Fortress Yayra Aku

**Affiliations:** 1 Department of Epidemiology and Biostatistics, Fred N. Binka School of Public Health, University of Health and Allied Sciences, Hohoe Campus, Hohoe, Ghana; 2 Hohoe Municipal Health Directorate, Ghana Health Service, Hohoe, Ghana; 3 Jasikan Municipal Heath Directorate, Ghana Health Service, Jasikan, Ghana; University of Michigan, UNITED STATES OF AMERICA

## Abstract

Vaccine hesitancy has been a long-standing concern globally and emerged during the COVID-19 pandemic among healthcare personnel. This study assessed the prevalence and determinants of vaccine hesitancy among healthcare personnel at the Volta Regional Hospital in Ghana. A cross-sectional study was conducted among 419 randomly selected healthcare personnel to collect sociodemographic, individual-level, community-level, and health system-level data, including vaccine uptake. Multivariable logistic regression analysis was performed to assess determinants of COVID-19 vaccine hesitancy at a 95% confidence level. COVID-19 vaccine hesitancy was found in 105 (25.1%) of respondents. Increased odds of vaccine hesitancy were associated with having 6–10 years of work experience (AOR = 3.43 95% CI = 1.44–8.18), being a midwife (AOR = 2.72 95% CI = 1.15–6.46), belief that vaccination does not guarantee protection against re-infection (AOR = 5.21 95% CI = 1.79–15.22), fear of long-term effects of COVID-19 (AOR = 2.14 95% CI = 1.18–3.86), perception of increased adverse effects among vaccinated individuals (AOR = 2.79 95% CI = 1.37–5.66), and community perception of long-term health effects of vaccination (AOR = 2.69 95% CI = 1.26–5.78). Awareness that health facilities were available to manage adverse effects reduced the odds of hesitancy (AOR = 0.23 95% CI = 0.12–0.43). Our study found that one in four healthcare personnel in the Volta Regional Hospital reported hesitancy toward receiving the COVID-19 vaccine. Targeted risk communication and confidence-building strategies tailored to midwives and healthcare workers with longer years of service, including interventions aimed at improving perceptions of vaccine efficacy and safety, may enhance COVID-19 vaccine uptake.

## Introduction

COVID-19 has emerged as a global public health concern, with substantial impacts on morbidity and mortality worldwide [1]. The World Health Organization (WHO) officially declared COVID-19 a global pandemic on March 11, 2020. This declaration marked a significant milestone in recognizing the widespread and sustained transmission of the virus across multiple regions worldwide [2]. As of February 23, 2025, the pandemic resulted in more than 777 million confirmed cases and 7 million deaths [3], with Africa accounting for 9.6 million cases and more than 176,000 deaths [3]. Ghana recorded 172,334 confirmed cases of COVID-19, resulting in 1,463 fatalities [3].

To address this public health emergency, a multifaceted public health response was mobilized, emphasizing vaccination as an essential intervention in mitigating the impact of the pandemic. At the time of writing this paper, more than 13.6 billion COVID-19 vaccine doses had been administered globally, with 67% fully vaccinated [3]. In Africa, 646 million doses had been administered, and 33% had been vaccinated with a complete primary series [3]. In Ghana, out of 25.6 million vaccine doses administered, only 35% of people achieved full vaccination status [3]. In the Volta Region of Ghana, more than 980,000 vaccine doses had been administered with only 26% of persons who were fully vaccinated [4]. Examining the Hohoe Municipality, located in the Volta region, about 95,000 doses had been administered, and approximately 41% of the estimated population attained full vaccination status [4].

Given the global context of vaccination interventions and the significant progress made in vaccine distribution and accessibility, across regions, it is undeniable that vaccine apprehension remains a prominent and complex challenge even before the COVID-19 pandemic. Surveys conducted in 2021 revealed varying levels of willingness to receive COVID-19 vaccine [5]. This multiplicity in attitudes towards vaccination emphasises the critical need to address vaccine hesitancy by enhancing confidence in immunization services provided to the population. Heightened levels of COVID-19 vaccine hesitancy, which ultimately resulted in low vaccine uptake, posed significant threats to the fight against the pandemic. For instance, it undermined strategic plans targeted at reducing COVID-19-related morbidity and mortality and emphasized the urgency for targeted interventions such as community engagement and communication strategies [6–8].

Looking at how health systems were overwhelmed during the pandemic, the health and safety of healthcare workers (HCWs) and other hospital staff were prioritised through infection prevention and control (IPC) strategies and vaccination [9–12]. Nonetheless, governments were faced with COVID-19 vaccine hesitancy among HCWs [13,14]. The development presented dire consequences such as staff shortages, which culminated into suboptimal service delivery, staff ill-health, and poor quality of life of patients receiving care [15].

Based on their vulnerability, HCWs were potential reservoirs for disease transmission both in the healthcare setting [16–18] and the community [16,19] and the heightened risk translated into potential complications, hospitalizations, and fatalities [20]. There were substantial number of COVID-19 infections and deaths reported among HCWs globally [20] amidst the availability of vaccines, with over 150,000 infections reported in Africa [21]. As of January 2022, more than 4,700 COVID-19 infections among HCWs had been documented in Ghana [21] following the first wave which occurred between June and August 2020, and the second wave which occurred between January and February 2021. Similar reports were documented in the Hohoe municipality of Ghana with 20.9% COVID-19 cases reported among HCWs [22]. This development impacted the psychosocial wellbeing of HCWs, and the already overwhelmed health system [22].

Prioritising the vaccination of HCWs and other hospital staff by the Ministry of Health and Ghana Health Service was a step to mitigate the COVID-19 burden [23]. Nonetheless, anecdotal accounts suggested the presence of vaccine hesitancy within this population in the country, corroborated by empirical evidence from various regions across Africa [24,25]. With their extensive knowledge of the benefits derived from vaccination, HCWs are not immune to concerns and anxieties regarding vaccination [14], especially given how complex and unique the COVID-19 pandemic emerged. This is exemplified by historical events such as the temporary suspension of Ebola trials in Hohoe, (the same township the regional hospital is located), due to such concerns [26,27]. The Volta Regional Hospital functioned as one of the designated COVID-19 treatment centers in the Volta Region of Ghana, and considering that vaccine hesitancy among healthcare personnel has the tendency to negatively impact community perceptions, particularly among patients and family members, potentially leading to refusal or delays in the uptake of the COVID-19 vaccine, we deemed this study necessary [28]. Previous studies in Ghana assessed the determinants of COVID-19 vaccine hesitancy among healthcare workers [29–32]. Nonetheless, theoretical gaps exist in understanding vaccine hesitancy among this population with limited studies applying the health belief model in the subject area in the Ghanaian context [33]. We therefore assessed vaccine hesitancy and its determinants among healthcare personnel (HCP), in the Volta Regional Hospital (formerly called the Hohoe Municipal Hospital) in Ghana using an integrated health belief model (HBM) and the theory of planned behaviour (TPB).

### Theoretical framework

Numerous theoretical models and frameworks have been developed to explain the determinants of health behaviour adoption, including: Theory of Planned Behaviour [34], Health Belief Model [35], Anderson’s behavioural model [36], and Protection Motivation Theory [37]. Though the HBM has been the most common model applied to understand vaccination uptake behaviour [38–40], Okai et al. combined the HBM with the TPB in assessing the determinants of COVID-19 vaccine uptake among Ghanaian adults [23]. We therefore adapted this model in our study.

**Fig 1:**
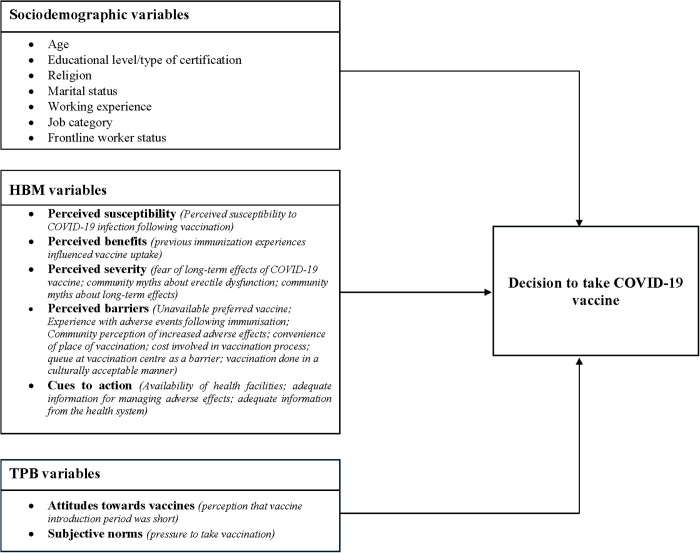
Conceptual framework of determinants of vaccine acceptance/hesitancy (adapted from Okai et al. [23].

The HBM theorizes that health behaviour-related decisions are dependent on one’s susceptibility to and perceived severity of illness, perceived benefits derived from engaging in preventive behaviour, perceived costs or barriers to the behaviour, and cues to action [35]. The theory hypothesizes that individuals tend to take preventive actions if they perceive themselves to be susceptible to illness; if they perceive the potential consequences to be severe; if they perceive that the option available to them will reduce their risk or severity, and if they realize that limited adverse features are associated with the health action [41]. Moreover, individuals’ decisions on health-related behaviour also tend to be influenced by internal and external environmental factors [42]. To account for environmental and social factors influencing health behaviour adoption, we integrated the HBM with the theory of planned behaviour (TPB). TPB hypothesises that, a person’s intention to engage in a behaviour is based on three functions namely: attitude, subjective norms, and perceived behavioural control [34].A person’s attitude reflects the degree to which they have a favourable assessment or otherwise of the action of interest [34], while subjective norms reflect beliefs an individual has that people who matter to him such as friends would endorse the behaviour or otherwise [43]. The third element, which is the perceived behavioural control reflects how the individual perceives that engaging in the behaviour will be simple or with difficulty [44]. However, given that factors such as the availability of vaccines, timing, cost, and prioritisation of vaccination was not within the HCP’s power, it was not considered in this study, similarly to what was done in a previous study [23]. Aside from the integrated HBM and TPB framework, previous studies have shown that sociodemographic characteristics such as age, gender, and type of healthcare worker [45–47] also influence the decision to take up vaccines (Fig 1).

## Materials and methods

### Study design, setting, and period

**Fig 2:**
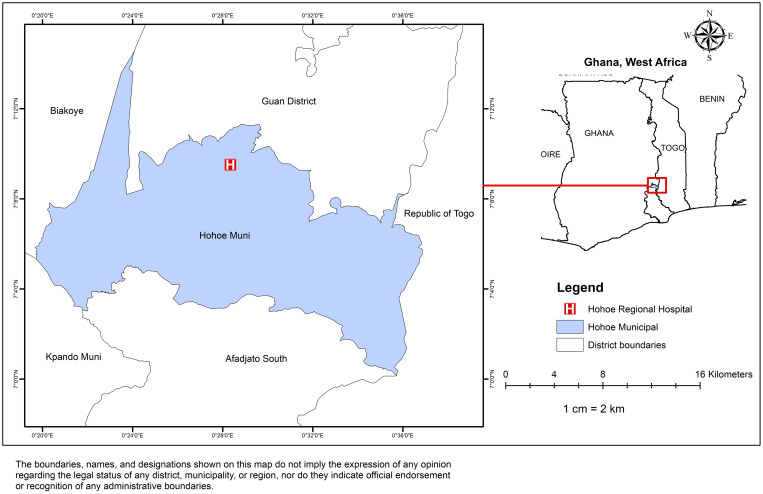
Map showing Volta Regional Hospital in the Hohoe Municipality, Ghana (The base layer shapefile was obtained from GADM (https://gadm.org/download_country.html) and visualized using ArcMap 10.4. GADM permits academic use, including publication of maps in open-access journals under CC-BY licenses. License terms available at https://gadm.org/license.html).

An institutional-based cross-sectional study design was conducted in the Volta Regional Hospital located within the Volta region in Ghana in October, 2022. The regional hospital is a referral hospital located in the Volta Region of Ghana (as shown in Fig 2), which provides comprehensive health services for both urban and rural populations surrounding the municipality. The facility attends to an estimated 112,000 out-patient department (OPD) clients annually. The hospital currently has a bed capacity of 178 with a staff strength of about 467 staff, including casual staff.

### Study population and eligibility criteria

The study population included all HCP who were working in the Volta Regional Hospital during the study period. All staff categories (clinical and non-clinical) were eligible to participate in the study.

### Sample size determination

The sample size was computed using the single population proportion formula [48], $n=\frac{{\left(z_{\alpha /2}\right)}^{2}\times p(1-p)}{d^{2}}$ with the following assumptions: estimated prevalence of COVID-19 vaccine hesitancy among general population in Ghana (p = 55%) [49]; Confidence level at 95% (Zα/2) = 1.96, a margin of error (d) = 0.05 and non-response rate = 10%. The minimum sample size determined was 419.

### Sampling procedure

Simple random sampling was employed to recruit respondents for the study. This was achieved by assigning a distinct code to the list of all HCP obtained from the human resource department of the hospital. The unique codes were then written individually on pieces of paper, concealed, and gathered into an opaque box. An independent individual was assigned the task of picking one paper at a time and recording the selected unique number. After each selection, the box was shaken, and the next paper was chosen until the 419^th^ paper. These selections were made without replacement. This ensured that every individual within the study population had an equal opportunity to be recruited into the study.

### Operational definitions

#### COVID-19 vaccine hesitancy.

For this study, respondents were defined as COVID-19 vaccine-hesitant if they had delayed in accepting or had refused the COVID-19 vaccine despite availability of vaccination services at the designated vaccination posts or health facility where they worked. This definition was based on the Strategic Advisory Group of Experts on Immunization (SAGE) working group on vaccine hesitancy [50].

#### Healthcare personnel.

Refers to all categories of staff, including both clinical and non-clinical staff, who worked at the Volta Regional Hospital during the study period. The definition is based on a similar population used in previous studies on COVID-19 vaccine hesitancy in Turkey and China [51,52].

### Study variables

#### Dependent variable.

The dependent variable in this study is vaccine hesitancy among HCP. It was measured as a binary outcome (Yes/No). (See S1 Table).

#### Independent variables.

- **Demographic variables:** Age group, sex, level of education, marital status, religion, work experience, frontline health worker status, and job category. (see S1 Table).
- **HBM and TPB constructs:** Seven constructs were utilized to examine COVID-19 vaccine hesitancy: perceived severity, perceived susceptibility, perceived benefits, perceived barriers, cues to action, attitude toward vaccination, and subjective norms. Data were collected on specific variables categorized as individual-level, health system-level and community-level characteristics and aligned with each construct to facilitate the analysis: **Individual level variables**: (1) Previous COVID-19 diagnosis: Whether the respondent had ever tested positive for COVID-19 (Yes/No). (2) Experience with adverse events following immunisation (AEFI): Whether the respondent had experienced any AEFI in the past (Yes/No). (3) Previous immunisation experiences influenced vaccine intake: Whether past experiences with vaccination affected willingness to take the COVID-19 vaccine (Yes/No). (4) Perceived susceptibility to COVID-19 infection following vaccination: Whether the respondent believed they could still contract COVID-19 after vaccination (Yes/No). (5) Fear of long-term effects of the COVID-19 vaccine: Whether the respondent expressed concern about potential long-term adverse effects (Yes/No). (6) Perception that vaccine introduction period was short: whether respondents perceived that the duration for manufacturing, approving and distributing vaccines was rather short (Yes/No). **Health system level variables:** (7) Availability of health facilities for managing adverse effects: Whether respondents believed the health system was adequately equipped to manage AEFIs (Yes/No). (8) Availability of preferred vaccine type: Whether the preferred vaccine was available at the vaccination site (Yes/No). (9) Pressure to take vaccine: Whether respondents felt compelled to accept vaccination irrespective of personal beliefs or readiness. **Community level variables:** (10) Community perception on erectile dysfunction and COVID-19 vaccination: Whether the respondent reported a common community belief that the vaccine causes erectile dysfunction (Yes/No). (11) Community perception of long-term health effects: Whether people in the community believed the vaccine causes long-term health issues, based on widely shared local myths or misinformation (Yes/No). (12) Community perception of increased adverse effects: Whether there was a belief in the community that the COVID-19 vaccine causes more frequent or severe side effects than other vaccines (Yes/No) (see Fig 1).

### Data collection instrument and method

The purpose of the study was explained before a standard set of paper-based questionnaires was administered to respondents between 1^st^ and 31^st^ October, 2022. In a face-to-face interview, data were collected on sociodemographic, individual-level, health system-level, community-level factors and vaccine hesitancy. The questionnaire items were developed based on operational definitions derived from existing literature on vaccine hesitancy. It was pretested with five participants to assess clarity and internal consistency. Reliability testing using Cronbach’s alpha was conducted separately for items under individual, health system, and community factors, with scale reliability coefficients of 0.79, 0.84, and 0.83 respectively.

### Data management and analysis

Data from the hardcopy questionnaires were entered into electronic KoboCollect forms and exported to Stata version 16 for cleaning and analysis. Descriptive statistics including frequencies and percentages were computed for categorical variables. Multivariable logistic regression was conducted to assess determinants of COVID-19 vaccine hesitancy. All variables that were significant at p < 0.05 in the bivariate analysis were included. In addition, important sociodemographic determinants such as age, sex, education, marital status and religion were included irrespective of their significance based on existing literature to account for potential confounding. Adjusted odds ratios (AOR) with 95% confidence intervals and p-values were reported. Statistical significance was set at p < 0.05. Only significant determinants were presented in the main results table with non-significant variables noted in the table footnote. Model robustness was assessed using fit statistics. McFadden R squared was 0.273 and McKelvey and Zavoina R squared was 0.420 which indicates an acceptable model fit. Variance inflation factors were all below 5 indicating no multicollinearity among predictors.

### Ethical considerations

Ethical approval was obtained from the Research Ethics Committee (REC) of the University of Health and Allied Sciences, Ghana (UHAS-REC B.10[078]21-22). Written permission was also obtained from the management of the Volta Regional Hospital with respondents providing written informed consent before recruitment into the study. Participation was voluntary and respondents were made aware they could withdraw anytime during the interview. Privacy and confidentiality were assured by conducting interviews in secluded places and coding questionnaires to anonymize personal information of respondents. To minimize the risk of contracting COVID-19, measures were implemented to mitigate this risk including correct and consistent face mask usage and observing a one-meter social distancing protocol during the entire interview process. The study was conducted according to the Declarations of Helsinki.

## Results

### Sociodemographic characteristics

Table 1 presents the sociodemographic characteristics of HCP. A 100% response rate was obtained (419 out of 419 selected respondents participated in the study) (See S1 Data). Of the 419 HCP interviewed, the mean age was 32.55 (± 5.78) years, with over half, 263 (64.0%), belonging to the age group 30–39 years. Approximately half, 202 (48.2%) of the respondents were males, while the majority 355, (84.7%) had attained tertiary level education. Most, 308 (73.5%) of the respondents identified as Christians, while less than a quarter, 79 (18.9%) were Muslims. Regarding work experience, more than half, 254 (60.6%) had worked for 5 years or less.

**Table 1:** Sociodemographic characteristics of HCP in the Volta Regional Hospital, Hohoe, Ghana, December 2022.

Variables	Frequency (N = 419)	Percentage (%)
Mean age (years) ± S.D	32.55 ± 5.78	
**Age group (years)**		
20-29	105	25.1
30-39	268	64.0
≥40	46	10.9
**Sex**		
Male	202	48.2
Female	217	51.8
**Educational level**		
Primary	22	5.3
Secondary	42	10.0
Tertiary	355	84.7
**Religion**		
Christianity	308	73.5
Islam	79	18.9
Traditional	32	7.6
**Marital status**		
Single	210	50.1
Married	209	49.9
**Working experience (years)**		
1-5	254	60.6
6-10	77	18.4
11 and above	88	21.0
**Job category**		
Nurse	169	40.3
Paramedics	198	47.3
Midwife	52	12.4
**Frontline health worker**		
Yes	224	53.5
No	195	46.5

S.D – Standard deviation

### Prevalence of COVID-19 vaccine

**Fig 3:**
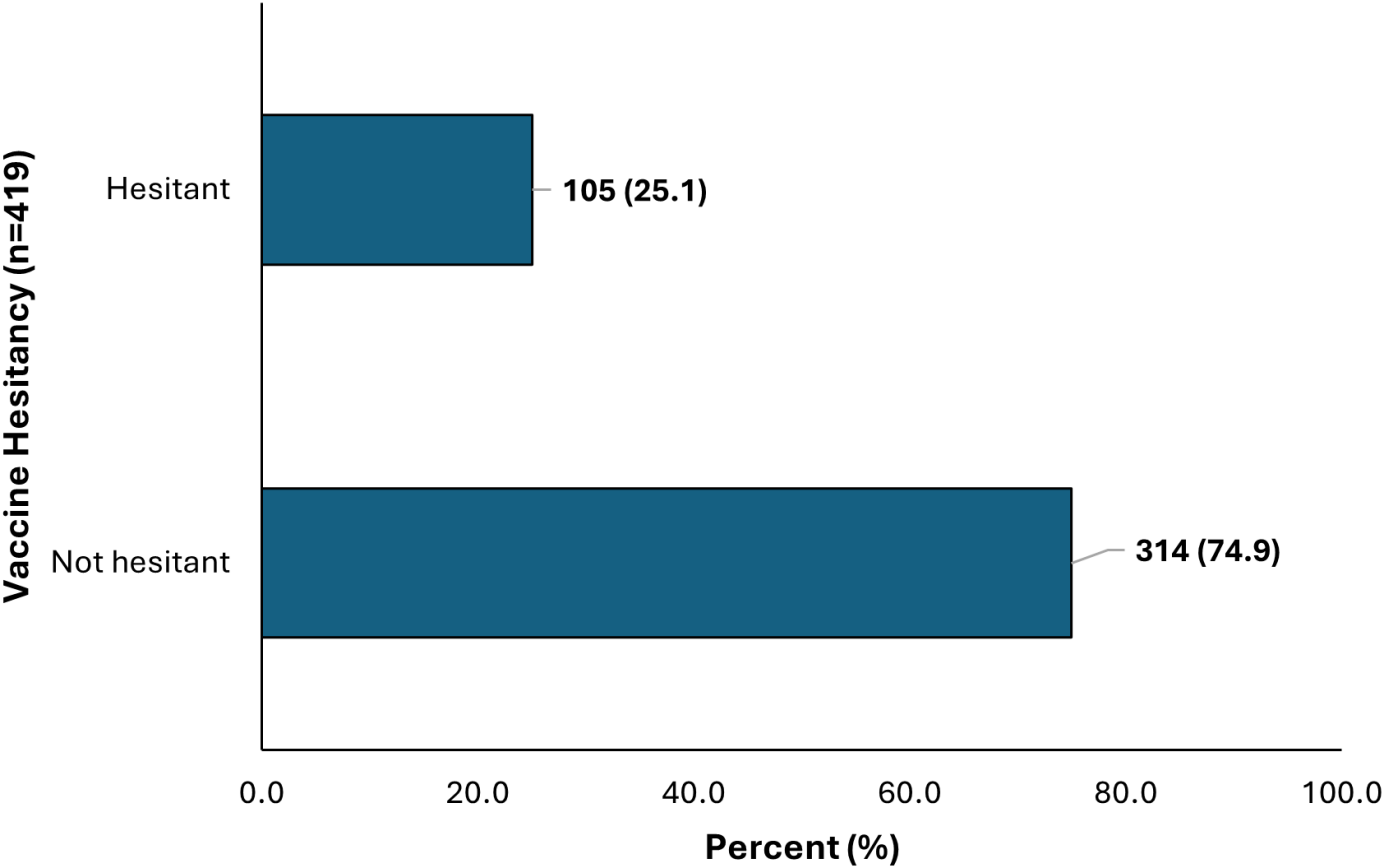
Prevalence of vaccine hesitancy among HCP in the Volta Regional Hospital, Hohoe, Ghana, December 2022.

Fig 3 shows the prevalence of vaccine hesitancy among HCP. One-quarter (105, 25.1%) of the 419 HCWs refused or delayed COVID-19 vaccination, while majority 314, (74.9%) accepted COVID-19 vaccination.

### Bivariate analysis

Table 2 presents bivariate analysis of variables associated with vaccine hesitancy. In the analysis, individuals aged 40 years and above were more likely to be COVID-19 vaccine hesitant compared to those aged 20–29 years (COR: 2.83, 95% CI = 1.29–6.21). Additionally, respondents with more than 5 years of working experience were more likely to be hesitant than those with less than 6 years of experience (COR: 1.82, 95% CI = 1.05–3.17). Paramedics/Nonclinical staff (COR = 2.34, 95% CI = 1.41–3.90) and midwives (COR = 2.55, 95% CI = 1.26–5.2) had a higher odds of vaccine hesitancy compared to nurses. Similarly, HCP who believed vaccination did not prevent reinfection of COVID-19 were three times more likely to be vaccine hesitant compared to their counterparts (COR = 3.17, 95% CI = 1.22 – 8.24). We also observed that those who feared experiencing the long-term effects of COVID-19 disease were more likely to be hesitant compared to those who did not have that fear (COR = 2.92, 95% CI = 1.78 – 4.8).

**Table 2:** Association Between Selected Variables and COVID-19 Vaccine Hesitancy Among Healthcare Providers at the Volta Regional Hospital, Hohoe, Ghana, December 2022.

Variables	Vaccine Hesitancy			COR (95% CI)	p-value
	Hesitant (N = 105)	Not hesitant (N = 314)	Total (N = 419)		
**Demographic variables**					
**Age group (years)**					
30-39	70 (66.7)	198 (63.1)	268 (64)	1.71 (0.96–3.04)	0.068
>=40	17 (16.2)	29 (9.2)	46 (10.9)	**2.83 (1.29–6.21)**	0.009*
20-29	18 (17.1)	87 (27.7)	105 (25.1)	1	
**Sex**					
Female	48 (45.7)	169 (53.8)	217 (51.8)	0.72 (0.46–1.13)	0.151
Male	57 (54.3)	145 (46.2)	202 (48.2)	1	
**Level of education**					
Secondary	19 (18.1)	23 (7.3)	42 (10)	2.81 (0.87–9.03)	0.083
Tertiary	81 (77.1)	274 (87.3)	355 (84.7)	1.01 (0.36–2.81)	0.992
Primary	5 (4.8)	17 (5.4)	22 (5.3)	1	
**Marital status**					
Married	59 (56.2)	150 (47.8)	209 (49.9)	1.4 (0.9–2.19)	0.136
Single	46 (43.8)	164 (52.2)	210 (50.1)	1	
**Religion**					
Islam	2 (1.9)	55 (17.5)	79 (18.9)	1.51 (0.87–2.62)	0.14
Traditional	1 (1)	20 (6.4)	32 (7.6)	2.08 (0.97–4.46)	0.061
Christianity	6 (5.7)	239 (76.1)	308 (73.5)	1	
**Work experience (years)**					
6-10	27 (25.7)	50 (15.9)	77 (18.4)	**1.82 (1.05–3.17)**	0.033*
11 and above	20 (19)	68 (21.7)	88 (21)	0.99 (0.56–1.77)	0.983
1-5	58 (55.2)	196 (62.4)	254 (60.6)	1	
**Frontline health worker**					
Yes	51 (48.6)	173 (55.1)	224 (53.5)	0.77 (0.49–1.2)	0.247
No	54 (51.4)	141 (44.9)	195 (46.5)	1	
**Job category**					
Paramedics	61 (58.1)	137 (43.6)	198 (47.3)	**2.34 (1.41–3.9)**	0.001*
Midwife	17 (16.2)	35 (11.1)	52 (12.4)	**2.55 (1.26–5.2)**	0.01*
Nurse	27 (25.7)	142 (45.2)	169 (40.3)	1	
**Individual- level variables**					
**Previous COVID-19 Diagnosis**					
Yes	15 (14.3)	52 (16.6)	67 (16)	0.84 (0.45–1.56)	0.582
No	90 (85.7)	262 (83.4)	352 (84)	1	
**Previous experience with adverse effects following immunization**					
Yes	34 (32.4)	72 (22.9)	106 (25.3)	1.61 (0.99–2.62)	0.055
No	71 (67.6)	242 (77.1)	313 (74.7)	1	
**Previous experience influenced vaccine intake**					
Yes	26 (24.8)	92 (29.3)	118 (28.2)	0.79 (0.79–1.32)	0.371
No	79 (75.2)	222 (70.7)	301 (71.8)	1	
**Confidence in COVID-19 vaccine effectiveness**					
Yes	79 (75.2)	254 (80.9)	333 (79.5)	0.72 (0.42–1.21)	0.221
No	26 (24.8)	60 (19.1)	86 (20.5)	1	
**Vaccination unacceptance on religious grounds**					
Yes	4 (3.8)	21 (6.7)	25 (6)	0.55 (0.19–1.65)	0.259
No	101 (96.2)	293 (93.3)	394 (94)	1	
**Vaccine effectiveness prevents the need for wearing a nose mask**					
Yes	29 (27.6)	79 (25.2)	108 (25.8)	1.14 (0.69–1.87)	0.62
No	76 (72.4)	235 (74.8)	311 (74.2)	1	
**Perception of vaccination not guaranteeing reinfection**					
Yes	100 (95.2)	271 (86.3)	371 (88.5)	**3.17 (1.22–8.24)**	0.007*
No	5 (4.8)	43 (13.7)	48 (11.5)	1	
**Fear of long-term effect of COVID-19**					
Yes	79 (75.2)	160 (51)	239 (57)	**2.92 (1.78–4.8)**	<0.001*
No	26 (24.8)	154 (49)	180 (43)	1	
**Health system variables**					
**Perception of COVID-19 vaccine safety**					
Yes	79 (75.2)	258 (82.2)	337 (80.4)	0.66 (0.39–1.12)	0.123
No	26 (24.8)	56 (17.8)	82 (19.6)	1	
**Perception that vaccine introduction period was short**					
Yes	78 (74.3)	207 (65.9)	285 (68)	1.49 (0.91–2.45)	0.113
No	27 (25.7)	107 (34.1)	134 (32)	1	
**Convenience of place of vaccination**					
Yes	90 (85.7)	253 (80.6)	343 (81.9)	1.45 (0.78–2.67)	0.238
No	15 (14.3)	61 (19.4)	76 (18.1)	1	
**Availability of health facilities for managing adverse effects**					
Yes	55 (52.4)	237 (75.5)	292 (69.7)	**0.36 (0.23–0.57)**	<0.001*
No	50 (47.6)	77 (24.5)	127 (30.3)	1	
**Cost involved in the vaccination process**					
Yes	4 (3.8)	30 (9.6)	34 (8.1)	0.37 (0.13–1.09)	0.072
No	101 (96.2)	284 (90.4)	385 (91.9)	1	
**Adequate information from the health system**					
Yes	82 (78.1)	248 (79)	330 (78.8)	0.95 (0.55–1.62)	0.848
No	23 (21.9)	66 (21)	89 (21.2)	1	
**Queue at vaccination centre as a barrier**					
Yes	10 (9.5)	18 (5.7)	28 (6.7)	1.73 (0.77–3.88)	0.183
No	95 (90.5)	296 (94.3)	391 (93.3)	1	
**Availability of preferred choice of vaccines at vaccination centre**					
Yes	60 (57.1)	237 (75.5)	297 (70.9)	**0.43 (0.27–0.69)**	<0.001*
No	45 (42.9)	77 (24.5)	122 (29.1)	1	
**Vaccination done in cultural acceptance of manner**					
Yes	84 (80)	271 (86.3)	355 (84.7)	0.63 (0.36–1.13)	0.122
No	21 (20)	43 (13.7)	64 (15.3)	1	
**Pressure to take vaccination decision**					
Yes	58 (55.2)	118 (37.6)	176 (42)	**2.05 (1.31–3.21)**	0.002*
No	47 (44.8)	196 (62.4)	243 (58)	1	
**Community variables**					
**Perception that vaccination causes erectile dysfunction**					
Yes	11 (10.5)	34 (10.8)	45 (10.7)	0.96 (0.47–1.98)	0.92
No	94 (89.5)	280 (89.2)	374 (89.3)	1	
**Community perception of long-term health effects in vaccinated individuals**					
Yes	83 (79)	145 (46.2)	228 (54.4)	**4.4 (2.61–7.39)**	<0.001*
No	22 (21)	169 (53.8)	191 (45.6)	1	
**Perception of increased vaccine adverse effects among vaccinated individuals**					
Yes	68 (64.8)	95 (30.3)	163 (38.9)	**4.24 (2.66–6.76)**	<0.001*
No	37 (35.2)	219 (69.7)	256 (61.1)	1	

COR=Crude odds ratio; CI=Confidence interval; *p-value<0.05

Concerning health system factors, HCP with the awareness that health facilities managed adverse effects following COVID-19 vaccinations were less likely to be hesitant (COR = 0.36, 95% CI = 0.23 – 0.57). Furthermore, availability of the preferred vaccine choice was also significantly associated with lower likelihood for vaccine hesitancy (COR = 0.43, 95% CI = 0.27–0.69). Furthermore, those who felt pressured to decide to vaccinate were twice as likely to be hesitant (COR = 2.05, 95% CI = 1.31–3.21) than their counterparts. Findings showed that HCP from communities perceiving long-term health effects in vaccinated individuals were 4.4 times more likely to be hesitant to vaccinate compared to those without such perceptions (COR = 4.4, 95% CI = 2.61–7.39). Likewise, individuals who perceived increased adverse effects following COVID-19 vaccinations were four times more likely to be hesitant (COR = 4.24, 95% CI = 2.66–6.76) than their counterparts.

### Multivariable analysis

Table 3 presents the multivariable analysis of factors independently associated with COVID-19 vaccine hesitancy among HCP. Having 6–10 years of work experience (AOR = 3.43, 95% CI = 1.44–8.18) and being a midwife (AOR = 2.72, 95% CI = 1.15–6.46) were associated with increased odds of hesitancy. Respondents who believed that vaccination does not guarantee protection against re-infection were also more likely to be hesitant (AOR = 5.21, 95% CI = 1.79–15.22). Those who reported fear of long-term side effects of the vaccine had increased odds of hesitancy (AOR = 2.14, 95% CI = 1.18–3.86). Similarly, individuals who reported that their communities believed in long-term health problems resulting from COVID-19 vaccination were more likely to be hesitant (AOR = 2.69, 95% CI = 1.26–5.78). The belief that vaccinated individuals experience increased adverse effects was also associated with greater hesitancy (AOR = 2.79, 95% CI = 1.37–5.66). On the other hand, respondents who reported the availability of health facilities for managing adverse effects were significantly less likely to be hesitant (AOR = 0.23, 95% CI = 0.12–0.43).

**Table 3:** Determinants of vaccine hesitancy among Healthcare Personnel in the Volta Regional Hospital, Hohoe, Ghana, December 2022.

Predictors	AOR (95% CI)	p-value*
Having 6–10 years work experience	3.43 (1.44–8.18)	**0.006**
Being a midwife	2.72 (1.15–6.46)	**0.023**
Believing that vaccination does not guarantee protection against re-infection	5.21 (1.79–15.22)	**0.003**
Fear of long-term effects of COVID-19	2.14 (1.18–3.86)	**0.012**
Perceiving increased adverse effects among vaccinated individual	2.79 (1.37–5.66)	**0.005**
Community perception of long-term health effects of vaccination	2.69 (1.26–5.78)	**0.011**
Availability of health facilities for managing adverse effects	0.23 (0.12–0.43)	**<0.001**

AOR=Adjusted odds ratio; CI=Confidence interval; *p-value<0.05

Only variables with statistically significant adjusted associations in final model (p < 0.05) are shown in the table. The following variables were included in the model but did not reach significance: age group, sex, education, marital status, religion, work experience (11 + years), job category (paramedics), availability of preferred vaccine and pressure to vaccinate.

## Discussion

The study assessed COVID-19 vaccine hesitancy and its determinants among healthcare personnel (HCP) at the Volta Regional Hospital of Ghana. We found vaccine hesitancy of 25.1% with associated determinants spanning sociodemographic, individual-level, health system level, and community-level factors. These factors closely align with the constructs of the HBM model, which is a widely applied theoretical framework for understanding health behaviours, including vaccination intentions and hesitancy [53–55].

Our findings showed a low level of hesitancy (25.1%) compared to 60.7% [32], and 40.7% [31] observed in previous studies in Ghana. Additionally, higher rates of vaccine hesitancy were observed in studies conducted elsewhere, including Sudan (36.2%) [56], Cameroon (54.6%) [57], Congo (72.3%) [25] and Sub-Saharan Africa (pooled prevalence of 46%) [58]. The low rate observed in our study could be attributed to the period in which the study was conducted, following at least two waves of the pandemic in Ghana. This temporal aspect is in line with understanding the perceived severity and cues to action constructs of the HBM [59,60]. During the initial phases of the pandemic, many people were sceptical about the rapid development and introduction of vaccines as a response strategy. This could potentially increase their perceived barriers to vaccination [59]. However, experiencing the direct impact of pandemic waves could increase an individuals’ perceived susceptibility to the disease, and the perceived severity of the disease, hereby enhancing vaccine acceptance as the threat becomes more salient and personally relevant [61,62].

Most, if not all, previous studies compared to our study were conducted during the initial phases of the pandemic. During that period, several people may have been overwhelmed with conspiracy theories and myths and therefore more likely to be hesitant towards vaccination. Some schools of thought believe that vaccine hesitancy has been powered by conspiracy theories, particularly, through social media [63,64].

As the pandemic evolved, response strategies, particularly risk communication and community engagement, played a critical role in addressing low-risk perceptions and countering misinformation surrounding COVID-19 vaccines within communities, thereby contributing to increased vaccine acceptance [65]. In Ghana, several national COVID-19 vaccination campaigns (also termed as NaCVaDs), were instituted by the government to improve vaccine uptake among all persons, which could have resulted in the lower rate of hesitancy observed in this study. Nonetheless, the finding that one in four healthcare providers remained hesitant to receive the COVID-19 vaccine, despite experiencing two waves of the pandemic and ongoing governmental interventions, underscores persistent gaps in vaccine confidence and highlights the need for more targeted and effective strategies.

Conversely, lower hesitancy rates were also reported in previous studies conducted in Kenya (4%) [66], China (8.7%) [67], South Africa (9.9%) [68] and on the global population (18.4%) [69]. Regional and population differences coupled with differences in health systems; vaccination campaign strategies; and individual health-seeking behaviour could account for the observed variance. Evidence from polio vaccination campaigns indicates that community acceptance was significantly hindered by the influence of anti-vaccine groups, the spread of conspiracy theories, ineffective communication strategies, religious beliefs, and circulating rumours [70], while a qualitative study among ethnic minorities from four London general practices showed that getting the chance to deliberate concerns with healthcare workers, emphasizing incentives and advocacy through social influences improved COVID-19 vaccination uptake [71]. Furthermore, a scoping review revealed that while the determinants of COVID-19 vaccination uptake may be widespread, segregated barriers may be linked to various target groups [72].

Our study found sociodemographic characteristics, susceptibility and severity perceptions, and health facilities’ preparedness as determinants of COVID-19 vaccine hesitancy, which was consistent with previous studies [64,73–77]. We observed that HCWs with longer working experience were more likely to refuse vaccination against COVID-19. Kwok and colleagues also observed that more experienced HCPs are less likely to vaccinate against COVID-19, and the less experienced staff are more likely to replicate this behaviour as they may consider the older ones as their role models [73]. Further, midwives showed higher odds of vaccine hesitancy compared to other HCPs. Similar patterns have been observed in previous studies, where specific professional roles showed varying levels of vaccine hesitancy. The observed differences may be attributable to varying levels of exposure to COVID-19 patients based on professional roles, prior experiences with vaccination, and differing degrees of trust in vaccine safety across professional groups [74,75,78,79].

Interestingly, perceived severity of COVID-19 in our study negatively influenced vaccine acceptance, as the fear of long-term effects of COVID-19 translated into higher odds of vaccine hesitancy. Conversely, the perceived severity of COVID-19 among healthcare personnel in studies in Ethiopia [80] and China [54] positively influenced vaccine uptake. The observed differences in our study may be attributed to concerns regarding the safety and effectiveness of COVID-19 vaccines, as well as negative perceptions of the vaccines, factors that have also been reported in a systematic review encompassing data from eight Sub-Saharan African countries [58].

Our study further suggests that perceptions of limited benefits from COVID-19 vaccination negatively influenced vaccine acceptance. This aligns directly with the HBM construct of perceived benefits, which posits that individuals are less likely to engage in a health behaviour if they do not believe it will reduce their risk or the seriousness of the threat [81]. Specifically, respondents who believed that vaccination does not protect against reinfection were more likely to exhibit hesitancy. This finding corroborates with other studies [64,76].

Similar to what we reported, the fear of side effects of COVID-19 vaccine (91.4%) in a study conducted in Egypt, was a key reason for vaccine hesitancy [75]. This is a prime example of the HBM construct of perceived barriers [81–83]. Individuals who perceive the side effects of vaccination to outweigh its benefits are more likely to exhibit vaccine hesitancy or refuse vaccination. Common concerns about side effects often cited in the literature include claims of infertility, altering human DNA, or containing tracking agents [61,84]. Such misinformation, often amplified through social media platforms including Twitter, Facebook, and YouTube [85], also erodes trust in official health authorities and scientific information, thereby diminishing the impact of credible cues to action [62,81]. The persistence of this concern highlights the need for tailored education and reassurance from health authorities on the safety of the vaccines. The negative influence of diminished perceptions of vaccination benefits was further emphasized by concerns about increased adverse events following vaccination, an issue consistently identified in various studies as a key driver of vaccine hesitancy [86,87]. Addressing these concerns through transparent and accurate communication about the risk-benefit profile of vaccines is essential in the fight against COVID-19 [75].

Regarding cues to action, respondents were less likely to be hesitant to vaccination on account of the availability of health facilities for managing adverse effects, suggesting that robust healthcare infrastructure can mitigate fears. It is evident the presence of accessible and reliable healthcare services functions serves as a strong cue to action, by both signalling support for the health behaviour and directly reducing perceived barriers to vaccination [60,61,88]. This finding is consistent with previous studies that, individuals’ willingness to get vaccinated increases when they believe that adverse effects will be promptly and effectively managed [89], on account that, they trust the overall safety of vaccination programs [90].

Moreover, community perception of long-term health effects increased the odds of vaccine hesitancy among respondents. It outlines the importance of community-level interventions and the influence of social networks on individual health behaviours [77,91], regardless of professional background, including healthcare workers. This is particularly important as healthcare workers serve as intermediaries between the health system and the population and their views may shape or reflect community attitudes. The Ghana Health Service led the extensive community engagement efforts due to its decentralized structure which culminated into increased vaccine acceptance among some community members. However, challenges such as insufficient logistics and widespread myths and misconceptions about vaccines led to distrust and vaccine hesitancy [92].

The study had important limitations to guide the interpretation of findings. First, the cross-sectional design of the study restricts our ability to infer causality; the observed associations may not necessarily imply a cause-and-effect relationship. Also, the reliance on self-reported data for vaccine hesitancy and its predictors could introduce biases such as social desirability bias or recall bias. Furthermore, the single study site used in our study excludes the generalizability of the findings to all healthcare personnel in Ghana. Finally, the sample size for the study was based on a vaccine hesitancy estimate from the general population due to the unavailability of subgroup-specific data at the time of designing the study. While we acknowledge that healthcare staff may exhibit different attitudes, using a general population estimate allowed us to avoid underpowering the study in the absence of specific pre-existing data for this subgroup. The application of the integrated HBM and TPB, however, provides a structured interpretation of the findings by linking the identified factors to established constructs of health behaviour. This provides a contextual understanding of the key barriers and facilitators of COVID-19 vaccine hesitancy, which is useful for stakeholders planning future vaccine strategies.

## Conclusion

This study adds to the literature on COVID-19 vaccine hesitancy by examining its prevalence and determinants among healthcare personnel in Ghana’s Volta Regional Hospital, reporting a comparatively lower hesitancy rate of 25.1%. This reduction is likely influenced by the timing of the study, following successive waves of the pandemic and intensified public health interventions. Despite this, persistent concerns, particularly among more experienced and specific professional groups, highlight the influence of systemic, psychological, and contextual factors such as fear of long-term effects, mistrust in vaccine safety, trust in the health system to manage adverse effects, and social influences. The findings underscore the importance of tailored, evidence-based communication strategies and the centrality of trust in healthcare systems. Vaccine hesitancy remains a dynamic issue, necessitating locally grounded and adaptive public health responses. Future vaccination programmes should prioritize strategies enshrined in the Centers for Disease Control and Prevention’s (CDC) Vaccinate with Confidence Framework to enhance healthcare personnel’s confidence in vaccine safety. This includes the development of evidence-informed communication initiatives that emphasize the long-term benefits of vaccination, reinforce confidence in the healthcare system’s capacity to manage potential adverse events, while proactively addressing misconceptions and fears, particularly during the pre-implementation and rollout phases. Moreover, tailored engagement strategies are warranted for more experienced HCPs and specific subgroups, such as paramedics and non-clinical staff, to ensure broader buy-in and sustained support for vaccination efforts across all tiers of the healthcare workforce.

## Supporting information

S1 Data A minimal dataset necessary to support the findings presented in the manuscript and to ensure the reproducibility of the results. (XLS)

S1 Table A summary of variable descriptions, value labels, and categorical classifications as used in the Stata analysis of the dataset supporting the findings presented in the manuscript. (DOCX)
