# Supplementary material for: Determinants of COVID-19 vaccine hesitancy among healthcare personnel in a secondary-level health facility in Ghana
Source: PLOS Glob Public Health. 2025 Sep 18;5(9):e0004980. doi: 10.1371/journal.pgph.0004980 (PMC12445576; doi:10.1371/journal.pgph.0004980)
Supplement: S1 Table — (DOCX) [file pgph.0004980.s002.docx]

# Variable Dictionary_COVID-19 vaccine hesitancy among healthcare professionals

| **SN** | **Variable Name** | **Category and Value Labels** |
| --- | --- | --- |
| 1 | agegrp | 1 = 20–29, 2 = 30–39, 3 = ≥40 |
| 2 | sex | 1 = Male, 2 = Female |
| 3 | edu | 2 = Primary, 3 = Secondary, 4 = Tertiary |
| 4 | marital | 1 = Single, 2 = Married |
| 5 | religion | 1 = Christianity, 2 = Islam, 3 = Traditional |
| 6 | working_years | 1 = 1–5, 2 = 6–10, 3 = ≥11 |
| 7 | frontline_worker | 0 = No, 1 = Yes |
| 8 | job_cat | 2 = Nurse, 3 = Paramedics, 4 = Midwife |
| 9 | covid_infection | 0 = No, 1 = Yes |
| 10 | adverse_reaction | 0 = No, 1 = Yes |
| 11 | reconsider_vaxx | 0 = No, 1 = Yes |
| 12 | confi_vaccine | 0 = No, 1 = Yes |
| 13 | relig_forbid | 0 = No, 1 = Yes |
| 14 | under_condition | 0 = No, 1 = Yes |
| 15 | guarant_mask | 0 = No, 1 = Yes |
| 16 | vaxx_infected | 0 = No, 1 = Yes |
| 17 | longterm_effect | 0 = No, 1 = Yes |
| 18 | safe | 0 = No, 1 = Yes |
| 19 | short_intro | 0 = No, 1 = Yes |
| 20 | convenient | 0 = No, 1 = Yes |
| 21 | treat_AEFI | 0 = No, 1 = Yes |
| 22 | cost | 0 = No, 1 = Yes |
| 23 | enough_infoHSS | 0 = No, 1 = Yes |
| 24 | queue_barrier | 0 = No, 1 = Yes |
| 25 | prefer_choice | 0 = No, 1 = Yes |
| 26 | accept_manner | 0 = No, 1 = Yes |
| 27 | push_vaccination | 0 = No, 1 = Yes |
| 28 | erect_dys | 0 = No, 1 = Yes |
| 29 | comm_longterm | 0 = No, 1 = Yes |
| 30 | vaccine_more_effects | 0 = No, 1 = Yes |
| 31 | outcome | 0 = Acceptance, 1 = Hesitance |
